# P378: Disinfectants policy in the hospitals -is it imperative???

**DOI:** 10.1186/2047-2994-2-S1-P378

**Published:** 2013-06-20

**Authors:** M Sri Ratnamani, R Rao

**Affiliations:** 1 Microbiology, Apollo hospitals Hyderabad India, Hyderabad, India

## Introduction

The effective use of disinfectants is part of a multibarrier strategy to prevent health-care–associated infections. In the absence of a regulatory body like EPA in India the disinfectants choice /use becomes more of individual based choice rather than scientific or evidence based. There is little or no awareness or impact of these chemicals on the health care workers(HCW) in the long term. Because efficacy testing cannot be performed by the laboratories belonging to small hospitals, one has to solely rely upon the literature provided by the manufacturer regarding the efficiency of the disinfectants. Almost all the manufacturers claim their disinfectant as a broad-spectrum antimicrobial agent suitable for diverse applications. [1]CDC recommends that persons or committees responsible for selecting disinfectants should be guided by information in the scientific literature^.^[2].

## Objectives

To standardize the process of disinfectants use throughout the hospital and ensure HCW safety.

## Methods

Hospital areas were divided it into critical, semi-critical and non-critical areas/public areas. Recommendations discussed in Infection prevention and control committee meeting. Policy distributed to the concerned areas.

## Results

Before Implementation- Varied disinfectants were being used throughout the hospitals There was no check on the type and dilution of disinfectants being used. After Implementation- Only recommended disinfectants are being used. There is a regular check on potency of disinfectants. Dilutions are done at the central Housekeeping desk.

## Conclusion

The policy has helped us in streamlining the cleaning and disinfection processes. There is stringent check on the type of disinfectants used in the hospital and any discrepancy or failure to adhere to the policy is brought to the attention of the ICT. An effective disinfectant policy can reduce the risk for infections associated with use of invasive and noninvasive medical and surgical devices.

## Disclosure of interest

None declared
